# Mean Scar Entropy by Late Gadolinium Enhancement Cardiac Magnetic Resonance Is Associated With Ventricular Arrhythmias Events in Hypertrophic Cardiomyopathy

**DOI:** 10.3389/fcvm.2021.758635

**Published:** 2021-11-17

**Authors:** Yang Ye, ZhongPing Ji, Wenli Zhou, Cailing Pu, Ya Li, Chengqin Zhou, Xiuhua Hu, Chao Chen, Yaxun Sun, Qi Huang, Wenjuan Zhang, Yu'e Qian, Hong Ren, Feidan Yu, Chenyang Jiang, Yankai Mao, Bei Wang, João B. Augusto, Dongwu Lai, Hongjie Hu, Guo-sheng Fu

**Affiliations:** ^1^Department of Cardiology, Sir Run Run Shaw Hospital, College of Medicine, Zhejiang University, Hangzhou, China; ^2^Key Laboratory of Cardiovascular Intervention and Regenerative Medicine of Zhejiang Province, Hangzhou, China; ^3^Institute of Graphics and Image, School of Computer Science and Technology, Hangzhou Dianzi University, Hangzhou, China; ^4^Department of Radiology, Sir Run Run Shaw Hospital, Zhejiang University School of Medicine, Hangzhou, China; ^5^Department of Cardiovascular, Zhejiang Integrated Traditional and Western Medicine Hospital (HangZhou Red Cross Hospital), Hangzhou, China; ^6^Department of Information Technology, Sir Run Run Shaw Hospital, Zhejiang University School of Medicine, Hangzhou, China; ^7^Department of Cardiac Echocardiology, Sir Run Run Shaw Hospital, Zhejiang University School of Medicine, Hangzhou, China; ^8^Department of Cardiology, Hospital Professor Doutor Fernando Fonseca, Lisbon, Portugal; ^9^Institute of Cardiovascular Science, University College London, London, United Kingdom; ^10^Cardiac Imaging Department, Barts Heart Centre, St Bartholomew's Hospital, London, United Kingdom

**Keywords:** hypertrophic cardiomyopathy, ventricular arrhythmias, scar entropy, cardiac magnetic resonance, late gadolinium enhancement

## Abstract

**Background:** Ventricular arrhythmias are associated with sudden cardiac death (SCD) in hypertrophic cardiomyopathy (HCM). Previous studies have found the late gadolinium enhancement (LGE) on cardiac magnetic resonance (CMR) was independently associated with ventricular arrhythmia (VA) in HCM. The risk stratification of VA remains complex and LGE is present in the majority of HCM patients. This study was conducted to determine whether the scar heterogeneity from LGE-derived entropy is associated with the VAs in HCM patients.

**Materials and Methods:** Sixty-eight HCM patients with scarring were retrospectively enrolled and divided into VA (31 patients) and non-VA (37 patients) groups. The left ventricular ejection fraction (LVEF) and percentage of the LGE (% LGE) were evaluated. The scar heterogeneity was quantified by the entropy within the scar and left ventricular (LV) myocardium.

**Results:** Multivariate analyses showed that a higher scar [hazard ratio (HR) 2.682; 95% CI: 1.022–7.037; *p* = 0.039] was independently associated with VA, after the adjustment for the LVEF, %LGE, LV maximal wall thickness (MWT), and left atrium (LA) diameter.

**Conclusion:** Scar entropy and %LGE are both independent risk indicators of VA. A high scar entropy may indicate an arrhythmogenic scar, an identification of which may have value for the clinical status assessment of VAs in HCM patients.

## Introduction

Cardiac magnetic resonance imaging is a valuable tool for the risk stratification of sudden cardiac death (SCD) in hypertrophic cardiomyopathy (HCM). The current gold standard for the visualization of a scarred myocardium is the late gadolinium enhancement (LGE) during cardiac magnetic resonance imaging (CMR) (1) and previous studies have demonstrated an association between ventricular LGE and ventricular arrhythmias (VAs) (2–4). The presence and extent of left ventricular (LV) scarring may have predictive utility for VAs in HCM patients (5).

Myocardial fibrosis is a significant cause of arrhythmogenesis in HCM patients (3, 6). However, LGE can be observed in the majority of HCM patients. Electrophysiology studies have shown that HCM patients with malignant arrhythmias have increased electrical dispersion and inhomogeneity of intraventricular conduction, which may be related to myocardial fibrosis in HCM patients with malignant arrhythmias (7). The measurement of entropy is useful for the evaluation of the heterogeneity in fibrotic lesions (8). The measurements convert the uncertainty of the signal intensity (SI) into the uncertainty of the tissue composition by taking all the SI values from the LGE-CMR. Entropy is reflected in the image complexity as a set of completely white pixels that would have an entropy value of zero, but the value increases as the scarring image intensifies, enabling the evaluation of the scar complexity. Previous studies have correlated the spatial heterogeneity of fibrosis, as defined by entropy measurements, with VAs in other cardiomyopathies (9, 10). The current study aims to determine whether the quantification of (1) scar heterogeneity, quantified by the entropy within the scar, may be considered a marker for inhomogeneous scar composition and to assess any association with VAs, and (2) the entropy of the entire LV quantify inhomogeneous fibrosis and assess any association with VAs.

## Materials and Methods

### Study Population

**Figure 1:**
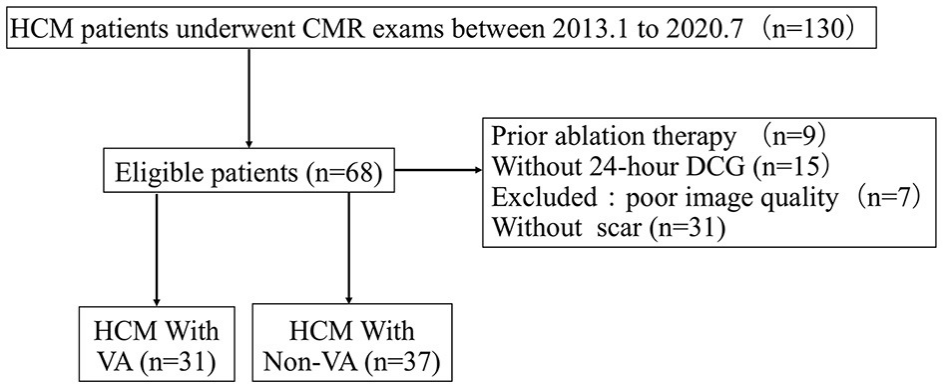
Flow diagram showing the selection and group of hypertrophic cardiomyopathy (HCM) with scar patients. CMR, cardiac magnetic resonance; DCG, dynamic electrocardiogram; HCM, hypertrophic cardiomyopathy; VA, ventricular arrhythmias.

Sixty-eight patients with a diagnosis of HCM (11), in whom scarring was observed by LGE-CMR at the Sir Run Run Shaw Hospital between January 2013 and January 2020 were retrospectively enrolled in the study (Figure 1). HCM was defined as having the maximal left ventricle wall thickness on CMR images ≥15 mm or as ≥13 mm, plus a documented family history of HCM according to the guidelines (11). The exclusion criteria included the following: prior septal reduction therapy; coronary artery disease; myocardial hypertrophy due to other causes (including aortic stenosis, myocardial storage diseases, hypertension); absence of a 24 h dynamic ECG (DCG) monitoring. The HCM patients without visible scarring and those for whom the LGE CMR quality was poor were also excluded. All the HCM patients had undergone clinical examination, CMR, and 24-h DCG. The participants in the study were divided into HCM with VA (31 cases) and HCM non-VA groups (37 cases). Evidence was collected from the patient medical records or *via* telephone. The study protocol was approved by the Sir Run Run Shaw Hospital ethics committee and complied with the Declaration of Helsinki.

### Assessment of VAs and SCD Risk Estimation

Ventricular arrhythmias included previous aborted cardiac arrest, documented sustained ventricular tachycardia (VT) (12), and non-sustained VT (NS-VT). VT was defined as the sustained ventricular arrhythmia over 100 heartbeats per minute when lasting longer than 30 s or requiring earlier intervention due to hemodynamic instability. Non-sustained VT was defined as the runs of ventricular beats between ≥3 beats lasting for <30 s with a heart rate (HR) of >100 bpm (12) during DCG. The SCD risk of the HCM patients was predicted using the ESC online HCM SCD Risk stratification score calculator (13). According to this risk model, the patients with an SCD score > 6% were considered as high risk, ≥4– <6% as intermediate risk, and <4% as low risk.

### Cardiac MRI Protocol and Analysis

Patients were imaged using 1.5 T CMR scanners [General Electric (GE) Healthcare, Chicago, Illinois or Magnetom Avanto Siemens, Erlangen, Germany]. All CMR images were acquired with electrocardiographic gating and breath-holding. Typical LGE CMR protocol parameters were applied: a repetition time of 4.8 ms, an echo time of 2.3 ms, and an inversion time of 200 adjusted to 300 ms. The LGE imaging was conducted 10 min after a cumulative intravenous injection of 0.2 mmol/kg of gadopentetate dimeglumine. A breath-hold segmented magnetization-prepared turbo gradient echo sequence was used with the inversion time chosen to null the normal myocardial signal. The images were visualized in 8–14 matchings short-axis (8 mm thick with 2 mm spacing) and three radial long-axis planes. The presence of LGE as a categorical value was first assessed by two radiologists who were blinded to the clinical data of the patients. The left ventricular ejection fraction (LVEF), left atrium (LA) diameter, maximal wall thickness (MWT), and %LGE were measured by standard volumetric techniques and analyzed with commercially available software (Circle Cardiovascular Imaging 42, version 5.10.1, Calgary, Canada). Late gadolinium enhancement was defined as the areas with adjusted gray-scale threshold ≥5 SD above the mean of the normal myocardium (14). The extent of the LGE (% LGE) was summarized and quantified as the percentage of the total left ventricular mass. Maximal wall thickness was defined as the greatest dimension at any site within the LV myocardium. Manual segmentation was performed by a CMR-trained cardiologist blinded to the patient identifiers and study endpoints. For inter-rater reliability, a second CMR-trained cardiologist performed a further manual segmentation, blinded to the results from the initial assessor.

The LV entropy and scar entropy were performed as previously described (9, 15) with the regions of interest drawn around all the visible LGE, carefully incorporating the scar border and excluding the surrounding myocardium. The quantification of the tissue inhomogeneity assumes that the varying SI values in LGE indicate the presence of tissues with different compositions. Tissue entropy, as an indication of inhomogeneity, was quantified for both the scar region and the entire LV myocardium. The borders of the endo-, epi-cardial, and scar regions were manually annotated. The range of the SI for each patient was normalized to a range of 0–1,024. For the LV entropy calculations, the SI values for all the pre-annotated pixels within the myocardium were used to evaluate the probability distributions for each patient. To calculate the entropy of the scar for each patient, the pixels within the pre-annotated scar areas were used to evaluate the probability distributions. Data loading and normalization were performed with PyRadiomics, and the scar-entropy and LV-entropy were calculated by Python using the following formula, derived from Shannon's formula:


$$\begin{array}{l} H\left(I\right)=-\overset{n}{\underset{i=1}{\sum }}p\left(I_{i}\right)log_{2}p\left(I_{i}\right) \end{array}$$

where $\overset{n}{\underset{i=1}{\sum }}\;$describes n SI values in the region of interest and p(Ii) is the probability of value Ii, which can be computed from the probability distribution. As the maximum intensity value was normalized to 1,024, the entropy ranges from 0 to 10, with 0 being a homogeneous distribution of a single intensity value, and 10 being a uniform distribution of varying intensity.

### Statistical Analysis

The continuous variables are presented as mean ± SD, and the categorical data are summarized as frequencies and percentages. The data that did not follow a normal distribution are presented as median and quartile (interquartile range). The differences in baseline characteristics between the patients were analyzed using a Student's *t*-test or Fisher exact test, as appropriate. Univariable and multivariable logistic regression analyses were used to study the relationship between age, LVEF, LA diameter, %LGE, scar entropy, LV entropy, and VAs. The HR was defined after the adjustment for the pre-determined potential confounders based on clinical relevance. The HR with 95% confidence intervals (CIs) are reported. The optimal cut-off values were determined by calculating the Youden index. Kaplan-Meier curves and log-rank tests were used to explore the association of the LGE and entropy with VAs. Receiver operator characteristic (ROC) curves were created and the value closest to the upper left corner determined the optimal sensitivity and specificity to discriminate between HCM patients with VAs. The inter-and intra-observer variabilities were expressed by the intra-class correlation coefficients. The inter-observer variability for quantitative scar and left ventricular entropy was assessed in a subset of 20 randomly selected CMR studies. Two readers (WLZ and CLP) independently quantitated the LGE without prior knowledge of the clinical data and were blinded to the previous results. For the intraobserver variability, two readers (CQ Z and ZP J) independently quantitated the entropy in a subset of 20 randomly selected CMR studies. The inter-rater agreement was evaluated using a Bland-Altman plot and linear regression analysis. All tests were two-tailed and *p* <0.05 was considered significant.

## Results

### Patient Characteristics

The baseline characteristics for all patients were presented in Table 1. A total of 68 HCM patients with scarring identified by both LGE-CMR and DCG were enrolled in our study (Mean age: 68.7 ± 12.2 years; 69.1% male). Twenty-seven patients were scanned with a Siemens 1.5-T scanner (Munich, Germany) and 41 patients were scanned with a 1.5-T GE scanner (Boston, MA, United States). Of the former, 48.2% (*n* = 13) had HCM with VA, and of the latter, 43.9 % (*p* = 0.81) (*n* = 18). In HCM with VAs, the 5-year SCD risk score was much higher among those with VA than those without VA {[5.2 (2.69, 4.80) vs. 1.80 (2.00, 2.00)]; *p* <0.001; Table 1}.

**Table 1:** Clinical characteristics in HCM patients with or without ventricular arrhythmias.

	**All HCM with** **LGE (*n* = 68)**	**HCM with** **VA (*n* = 31)**	**HCM without** **VA (*n* = 37)**	***p***
Mean age, years	54.5 ± 14.3	55.5 ± 13.5	53.8 ± 15.1	0.62
Male, *n* (%)	47 (69.1)	23 (74.2)	24 (64.9)	0.328
Hypertension, *n* (%)	26 (38.2)	12 (38.7)	14 (37.8)	0.941
Diabetes, *n* (%)	6 (8.8)	2 (6.5)	4 (10.8)	0.528
Creatinine(μmol/L)	76.9 ±18.1	80.7 ±18.9	73.8 ±17.2	0.15
Atrial fibrillation, *n* (%)	5 (7.3)	2 (6.5)	3 (8.1)	0.794
Syncope history, *n* (%)	9 (13.2)	6 (19.4)	3 (8.1)	0.173
Family history of SCD, *n* (%)	6 (8.8)	4 (12.9)	2 (5.4)	0.278
Obstruction present, *n* (%)	26 (38.2)	13 (41.9)	13 (35.1)	0.565
CMR findings				
LVEF %	67.2 ± 12.9	62.5 ± 15.5	71.2 ± 8.7	0.005
LA diameter, mm	38.8 ± 6.7	41.0 ± 6.0	36.9 ± 6.7	0.01
Mean MWT, mm	22.7 ± 5.6	24.3 ± 5.8	21.7 ± 4.6	0.04
%LGE	8.7 ± 8.6	13.6 ± 10.3	8.8 ± 8.5	<0.001
Mean LGE entropy	6.5 ± 1.1	6.8 ± 0.7	6.2 ± 1.3	0.01
Mean LV entropy	6.3 ± 1.1	6.5 ± 0.7	6.1 ± 1.3	0.127
Medications				
β-blockers, *n* (%)	46 (67.6)	21 (67.7)	25 (67.6)	0.988
Calcium antagonists, *n* (%)	21 (30.9)	9 (29.0)	12 (32.4)	0.762
ESC SCD risk				
% 5-year ESC SCD risk	3.4 ± 2.3	5.2 ± 2.2	1.8 ± 0.7	<0.001
High	6 (8.9)	6 (19.4)	0 (0)	
Intermediate	15 (22.1)	15 (48.4)	0 (0)	
Low	41 (60.3)	10 (32.3)	31 (100)	

*Values are mean ± SD or n (%). The p-values reflect comparison between 2 groups. HCM, hypertrophic cardiomyopathy; VA, ventricular arrhythmia; CMR, cardiac magnetic resonance; LVEF, left ventricular ejection fraction; LA, left atrium; LV, left ventricular; LGE, late gadolinium enhancement; SCD, sudden cardiac death*.

### Assessment of Entropy by CMR

The data for the left cardiac function and myocardial entropy data are summarized in Table 2 along with the respective patient age (55.5 ± 13.5 vs. 53.8 ± 15.1 years, *p* = 0.63). The mean entropy within the scar was significantly higher in the VA group (6.8 ± 0.7 vs. 6.1 ± 1.2, *p* = 0.004). There was no significant difference in the mean LV entropy between the two groups (Table 1). All the HCM patients had LGE detectable by CMR (Table 1). There was a weak association between the presence and extent of LGE and scar entropy (*r* = 0.287, *p* = 0.018) but none between LGE and LV entropy (*r* = 0.106, *p* = 0.398).

**Table 2:** Univariate and multivariate logistic regression analyses for predictors of ventricular arrhythmia in HCM patients with LGE-CMR.

	**Univariate logistic regression**	***p***	**Multivariate logistic regression**	***p***
	**OR, 95% CI**		**OR, 95% CI**	
Age (years)	1.018, 0.984–1.054	0.307		
MWT (mm)	1.120, 1.011–1.241	0.03	1.161, 1.011–1.333	0.034
Family history of SCD (yes vs.no)	0.386, 0.066–2.265	0.291		
Syncope (yes vs.no)	1.000, 1.000–1.000	0.551		
LA diameter	1.129, 1.037–1.228	0.005	1.104, 1.007–1.209	0.036
LVEF%	0.955, 0.915–0.996	0.032	0.986, 0.928–1.047	0.642
Obstruction presence (yes vs.no)	0.750, 0.281–2.002	0.566		
%LGE	1.255, 1.109–1.420	<0.001	1.251, 1.088–1.439	0.002
Scar entropy	2.870, 1.372–6.001	0.005	2.682, 1.022–7.037	0.039
LV entropy	1.557, 0.854–2.839	0.149		

*Values are mean ± SD or n (%). The p-values reflect a comparison between two groups with or without VA. HCM, hypertrophic cardiomyopathy; MWT, maximal wall thickness; SCD, sudden cardiac death; LA, left atrium; LVEF, left ventricular ejection fraction; LV, left ventricular; LGE, late gadolinium enhancement; %LGE, the percentage of late gadolinium enhancement (LGE) to left ventricular mass*.

### Assessment of VA in HCM Patients With LGE by CMR

Ventricular arrhythmias were documented in 31 patients (45.6%). Of these, 4 patients (12.9 %) had VA with aborted cardiac arrest, 7 (22.6 %) had sustained VT, and 20 (64.5%) had NS-VT. The HCM patients with VAs had poorer LVEF (62.2 ± 15.5% vs. 71.2 ± 8.7%; *p* = 0.005) and thicker MWT (24.3± 5.8 vs. 22.7 ± 5.7; *p* = 0.030) compared with those without VA.

The univariate analysis showed that the mean entropy within the scar, %LGE, MWT, LA diameter, and LVEF were all associated with the appropriate VA (Table 2). The CMR-derived parameter, higher scar entropy, is clearly associated with VA (HR: 2.870 per unit entropy; 95% CI: 1.372–6.001; *p* = 0.005) (Table 2). The multivariate analysis also related scar entropy with VA (HR: 2.682 per unit entropy; 95% CI: 1.022–7.037; *p* = 0.039; Table 2).

**Figure 2:**
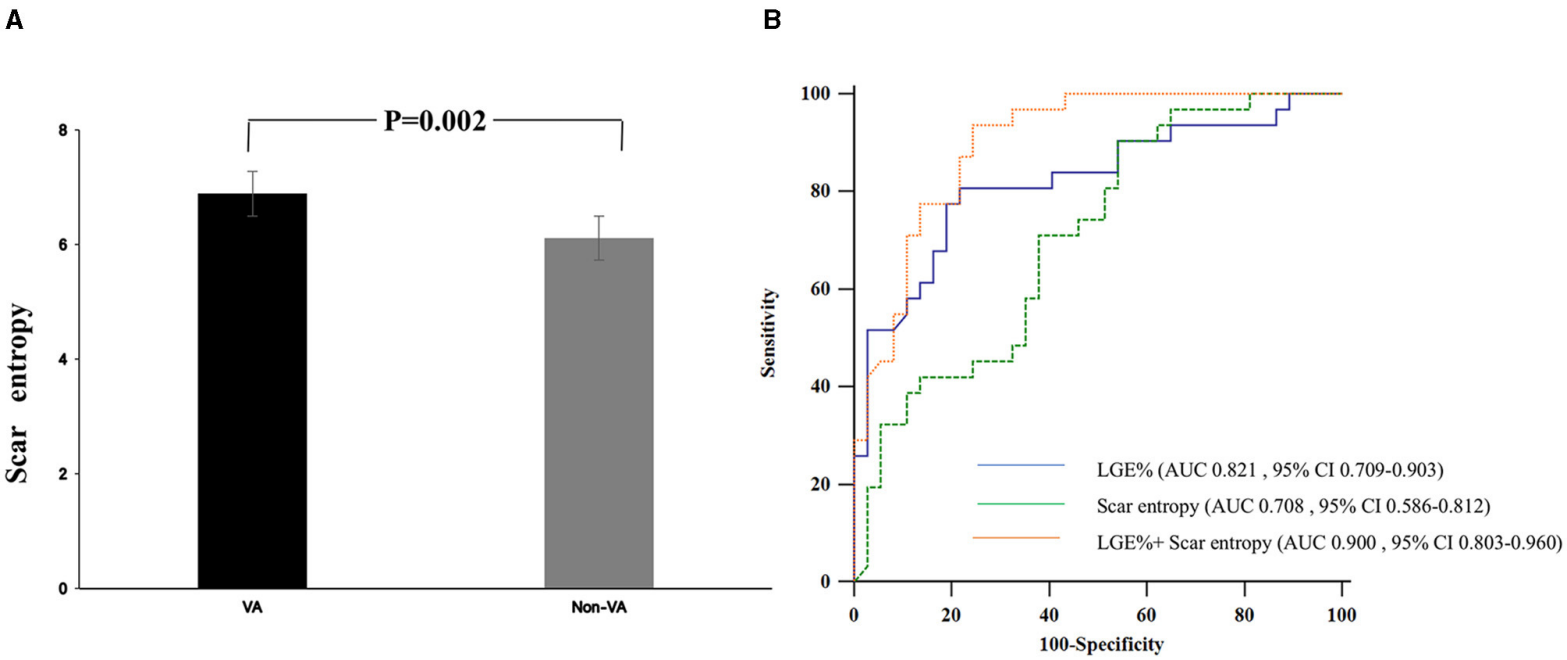
**(A)** Levels of scar entropy in HCM patients with or without ventricular arrhythmia (VA) (Groups VA and non-VA). **(B)** The receiver operator characteristic (ROC) curve of the scar entropy to predict the presence of VAs in the HCM cohort. AUC, area under the curve; CI, confidence interval; HCM, hypertrophic cardiomyopathy; VAs, Ventricular arrhythmias; The ROC curve of the percentage of the LGE (%LGE), scar entropy, and combined %LGE and scar entropy to predict the presence of VAs in the HCM cohort.

**Figure 3:**
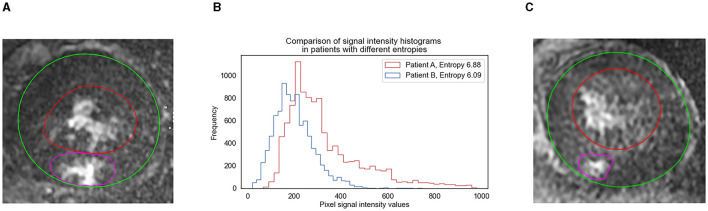
Scar entropy model and signal intensity (SI) histograms in two HCM subjects with or without VAs. The image demonstrates the uniformity of signal intensity in the scar, with the epicardial border in green and the endocardial border in red. The area shaded in red identifies the scar detected by the full width at the 5-SD method. **(A)** Image from an HCM patient who experienced VA events. **(C)** Image from an HCM patient who experienced non-VA events. **(B)** The histogram (middle) shows the probability distribution of the pixel signal intensities for both patients. Note the high frequency of pixels within a narrow range of SIs for patient B (blue line), suggesting little heterogeneity of the scar tissue and this patient had scar entropy of 6.09. Note the more dispersed distribution of SIs for patient A (orange line). The patient had scar entropy of 6.88, indicating a much more heterogeneous scar. Both patients were scanned using 1.5-T scanners.

There was no significant difference between %LGE and scar entropy by DeLong's test (*p* = 0.175). The efficiency of scar entropy in predicting the occurrence of VAs was evaluated by the ROC curve analysis (Figure 2). The green curve represents the scar entropy with a C-statistics value of 0.708 (95% CI: 0.586–0.812) and the blue curve represents the scar entropy with a C-statistics value of 0.821 (95% CI: 0.709–0.903; *p* = 0.175 vs. %LGE). The red curve represents the effect of adding entropy to the %LGE values and producing a better prediction of VAs in the HCM patients [area under the curve (AUC) = 0.900; 95% CI: 0.803–0.960] (*p* = 0.04 vs. %LGE; Figure 2). Figure 3 shows examples of the entropy model in two HCM subjects with or without VA. Patient A (orange line) had little heterogeneity of the scar tissue (in red) and this patient had a scar entropy of 6.09. Patient B (blue line) had a more dispersed distribution of SI and a scar entropy of 6.88.

**Figure 4:**
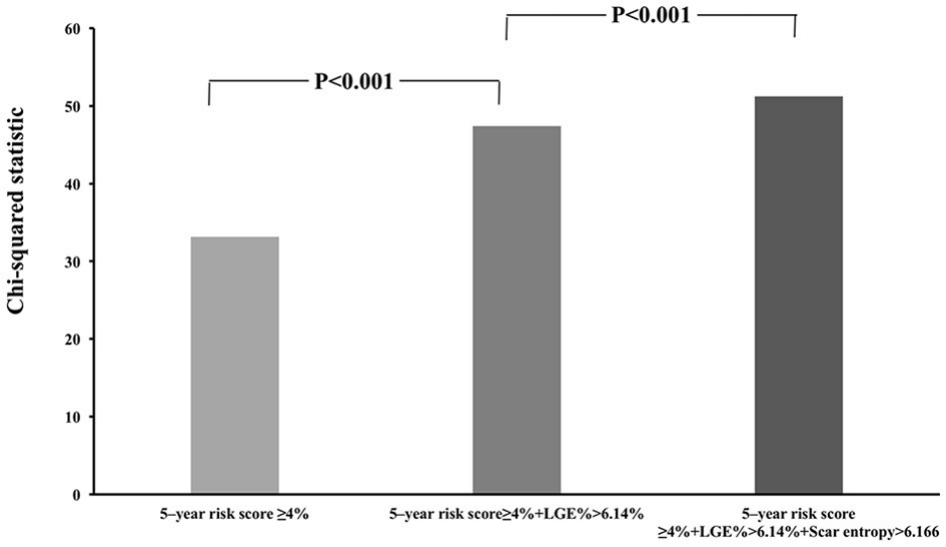
Incremental value of scar entropy >6.166 and LGE > 6.14% over the conventional 5-year sudden cardiac death (SCD) risk stratification score. The %LGE significantly improves the risk stratification of VAs when added to the 5-year SCD risk score ≥4% while scar entropy further significantly improved the %LGE-modified 5 year SCD risk stratification score.

The optimal cut-off values from the ROC analyses demonstrated that the patients with scar entropy values higher than 6.166 experienced VAs more frequently, with the sensitivity of 0.903% and the specificity of 0.459%. Similarly, the optimal cut-off values from the ROC analyses showed that the patients with %LGE higher than 6.14% had VAs more frequently. Patients with an ESC risk score higher than 4% (intermediate to high risk), combined with %LGE > 6.14% had significant risk stratification for VAs compared with those with a 5-year risk score ≥ 4% (*p* < 0.001; Figure 4). A scar entropy value of >6.166 further refined the % LGE-modified SCD Risk stratification score (*p* < 0.001 compared with the group of 5-year risk score ≥4%+%LGE > 6.14%; Figure 4).

At a median follow-up time of 25 months (IQR 13–13), no patients died. Among these, 12 of the HCM patients with VA had received implantable cardiac defibrillation (ICD) devices. During the follow-up, the median time of the VAs after CMR was 1 month (IQR 0–4.75). Among the HCM patients without VA, only one received an ICD for primary prevention.

### Intra- and Inter-observer Reproducibility

We found a good reproducibility of scar and LV entropy measurements for the intra- and inter-observer variability and the intraclass correlation coefficient (ICC) values for entropy were over 0.9. The summaries of the ICC values in both the intra- and inter-observer reproducibility are shown in Supplementary Figures 1A–D.

## Discussion

Our study demonstrated that the LGE-CMR-based calculations of entropy within the scar were associated with the occurrence of VA. The incorporation of the scar entropy values provides a significant refinement of the % LGE-modified 5-year SCD risk score. This modification has the potential to greatly assist the identification of high-risk HCM patients using LGE-CMR.

### CMR Findings Related to VAs in HCM Patients

Currently, available risk stratification indices only identify a limited proportion of HCM patients at risk of SCD. The current study used univariate analysis to show that the mean entropy within the scar, %LGE, MWT, LA diameter, and LVEF were all associated with the occurrence of VAs (Table 2). The HCM patients with VAs had thicker MWTs and larger LA diameters compared with those without (Table 1). Our study is consistent with the findings of previous studies which have linked MWT and LA diameter with VAs (16). The increased maximal left ventricular wall thickness is a marker of the risk for SCD in HCM (17). The left atrial size may also be used to predict adverse cardiac events in HCM patients (18, 19).

The current study showed that the HCM patients with VAs had worse LVEF by univariate analysis although no significant association could be shown by the multivariate analysis. The LVEF is not incorporated into the ESC risk model (13). Previous reports have indicated that ~3–4% of HCM patients have a reduced LVEF (20, 21). The low rate of this abnormality may be explained by the fact that hypertrophic myocardium pathology can produce normal or even higher LVEF (10). However, the early myocardial strain might be a predictor of worsening conditions in HCM patients (22). Adding LVEF to the ESC risk model is useful for further risk assessment of life-threatening arrhythmic events (23).

We demonstrated that a higher entropy within the scar was the CMR-derived parameter most closely associated with the occurrence of VAs (HR: 2.870 per unit entropy; 95% CI: 1.372–6.001; *p* = 0.005) (Table 2). Multivariate analysis confirmed this association (HR: 2.682 per unit entropy; 95% CI: 1.022–7.037; *p* = 0.039; Table 2). Using the optimal cut-off values from the ROC analyses, the patients with scar entropy values higher than 6.166 (log-rank *p* = 0.021) and %LGE higher than 6.14% (log-rank *p* < 0.001) had VAs more frequently. There was no significant difference between the %LGE and scar entropy by DeLong's test (*p* = 0.175). The efficiency of scar entropy in predicting the occurrence of VAs was further evaluated using ROC curve analysis (Figure 2). The green curve represents the scar entropy with a C-statistics value of 0.708 (95% CI: 0.586–0.812), the blue curve represents the scar entropy with a C-statistics value of 0.820 (95% CI: 0.586–0.812; *p* = 0.175 vs. %LGE) and the red curve represents the scar entropy combined with %LGE with a C-statistics value of 0.900 (95% CI: 0.803–0.906; *p* = 0.04 vs. %LGE). Using the optimal cut-off values from the ROC analyses, the patients with a scar entropy higher than 6.166 had VAs more frequently (sensitivity: 0.903% and specificity: 0.459%). A value for %LGE> 6.14% significantly refined the risk stratification for VAs when added to the conventional SCD Risk stratification score ≥4% (*p* < 0.001; Figure 4). The combination of %LGE with scar entropy >6.166 further refined the risk stratification, producing a performance that exceeded that of the SCD Risk stratification score modified by %LGE alone (*p* < 0.001).

Many new techniques and modifications of CMR require further research. Myocardial texture analysis helps in the detection of myocardial disease (24) and this could help in stratifying risk in HCM patients. In addition, myocardial T1 mapping and extracellular volume (ECV) fraction assist with HCM risk stratification (25) and T2 mapping has the potential to be a biomarker (26).

### LGE and Entropy in HCM Patients

Late gadolinium enhancement is an independent predictor of SCD in HCM patients (27) and the presence of LGE and its extent may be associated with a worse prognosis (5–8). However, current strategies for identifying high-risk patients are limited. Cardiac magnetic resonance with LGE can identify areas of myocardial fibrosis where life-threatening VAs originate (28). However, controversies remain regarding the independent prognostic importance of LGE-CMR in HCM since the presence of LGE may not always equate with the severity of fibrosis (6). Indeed, HCM patients with the same degree of LGE may have different outcomes.

The current study identified the novel marker, scar entropy, which refines the uncertainty in the LGE signal (8). Recent studies have shown that scar entropy correlates with VAs in other cardiomyopathies (9, 10)_._ The current study showed a very weak association between LGE and scar entropy and no association between LGE and LV entropy in HCM patients. Multivariate analysis identified the entropy within the scar but not that of the total LV myocardium as the LGE-derived parameter associated with the occurrence of VAs. As such, the entropy calculated two-dimensional LGE MR imaging seems to be a promising parameter to indicate the presence of an arrhythmogenic scar. The LGE extent is not a satisfactory predictor of risk due to the variable phenotype of HCM. The current retrospective study has shown that some patients with similar LGE extents experienced different outcomes. This may be partially explained by the heterogeneity of fibrosis which can be equated with scar entropy.

Entropy independently predicts adverse cardiac events and all-cause mortality in HCM patients (9, 29). Higher scar entropy has been associated with VAs, indicating the presence of an arrhythmogenic scar following myocardial infarction (9). Our findings show that scar entropy in HCM patients could further help to stratify the risk of patients at risk of VAs. The LGE volume and scar entropy in combination could refine the risk stratification for VAs. We have shown that the %LGE refined the risk stratification for VAs. We have shown that the %LGE refined the risk stratification for VAs when added to the conventional SCD risk stratification score and the addition of scar entropy provided a further refinement (*p* < 0.001).

The CMR assessments of LGE constitute a promising tool for SCD risk in HCM patients (30). However, there is little correlation between LGE and the degree of myocardial damage and risk (31–33), underscoring the need to improve risk stratification beyond the mere presence of LGE. LGE is associated with the occurrence and burden of VAs on Holter monitoring (2, 5, 34). Quantitative LGE analysis for a 5 SD threshold confirmed the correlation between entropy abnormalities and %LGE. A previous study has shown a linear correlation between the extent of LGE and the magnitude of risk with a value above 15% producing a two-fold increased risk in the otherwise “low risk” patients (6). However, the previous study cited above included only 50% of the patients with LGE by CMR. The present study adopted a cutoff value of 6.14% for the %LGE in the patients with VAs and all the HCM patients enrolled had LGE, thus accounting for some differences in findings.

We found that scar entropy was higher in the HCM patients with VAs and constituted an independent predictor of arrhythmias, indicating a continuum of risk assessed by this parameter. We suggest that a noninvasive identification of inhomogeneous scars and their evaluation by scar entropy is a superior tool to the mere presence and extent of LGE for risk stratification in HCM patients.

### Clinical Implications

Ventricular tachycardia is an important risk factor of SCD in large HCM studies and is most frequently assessed by Holter monitoring. However, this approach carries a risk of missing and underestimating events within the timescale available for registration. The present study shows that scar entropy could help assess the arrhythmic risk in HCM patients, assisting with the identification of high-risk individuals.

## Limitation

We acknowledge several limitations to our study. First, this study was retrospective, including relatively low numbers of HCM patients with both DCG and CMR results from a single medical center. Second, our 24 h monitoring period may be too short to accurately represent the overall picture of the arrhythmic burden of a patient making it quite limited in terms of follow-up. Though most patients had NS-VT and a high percentage had VAs in our HCM cohort, all had scarring identified by LGE-CMR. Third, risk stratification within these narrow parameters is quite difficult and challenging and further studies are needed to confirm the validity of the information contributed by our new technique. Future prospective investigations including larger cohorts with outcomes including VA and SCD are required to confirm our findings. Fourth, we did not get the information of the genetic testing results as this could also affect accurate risk stratification for a clinical diagnosis of HCM. Mapping techniques might add more values for risk stratification in HCM, which could be evaluated in future studies.

## Conclusion

The entropy used to quantify scar tissue in homogeneity within the scar was independently associated with VAs and therefore seems to be a promising marker of an inhomogeneous and arrhythmogenic scar in HCM patients.

## Data Availability Statement

The raw data supporting the conclusions of this article will be made available by the authors, without undue reservation.

## Ethics Statement

The studies involving human participants were reviewed and approved by Sir Run Run Shaw Hospital Ethics. The patients/participants provided their written informed consent to participate in this study.

## Author Contributions

DWL, HJH, and GSF conceived and designed the experiments. YY, ZPJ, WLZ, CLP, CQZ, XHH, CC, YXS, QH, WJZ, YEQ, HR, FDY, CYJ, YKM, and BW analyzed and interpreted the data. YY, ZPJ, and JBA wrote or edited the manuscript. All authors contributed to the article and approved the submitted version.

## Funding

This work was funded by the National Natural Science Foundation of China (Grant Number 81873908), Key Research and Development Program of Zhejiang Province (Grant Number 2019C03022), Medical Science and Technology Project of Zhejiang Province (Grant Number 2020KY220 and 2022506537), Chinese Medicine Research Program of Zhejiang Province (Grant Number 2020ZA083), and the funding from the Clinical Research Project of Zhejiang Medical Association (Number 2016ZYC-A28).

## Conflict of Interest

The authors declare that the research was conducted in the absence of any commercial or financial relationships that could be construed as a potential conflict of interest.

## Publisher's Note

All claims expressed in this article are solely those of the authors and do not necessarily represent those of their affiliated organizations, or those of the publisher, the editors and the reviewers. Any product that may be evaluated in this article, or claim that may be made by its manufacturer, is not guaranteed or endorsed by the publisher.
