# Supplementary figures and images for: Mean Scar Entropy by Late Gadolinium Enhancement Cardiac Magnetic Resonance Is Associated With Ventricular Arrhythmias Events in Hypertrophic Cardiomyopathy
Source: Front Cardiovasc Med. 2021 Nov 17;8:758635. doi: 10.3389/fcvm.2021.758635 (PMC8635716; doi:10.3389/fcvm.2021.758635)

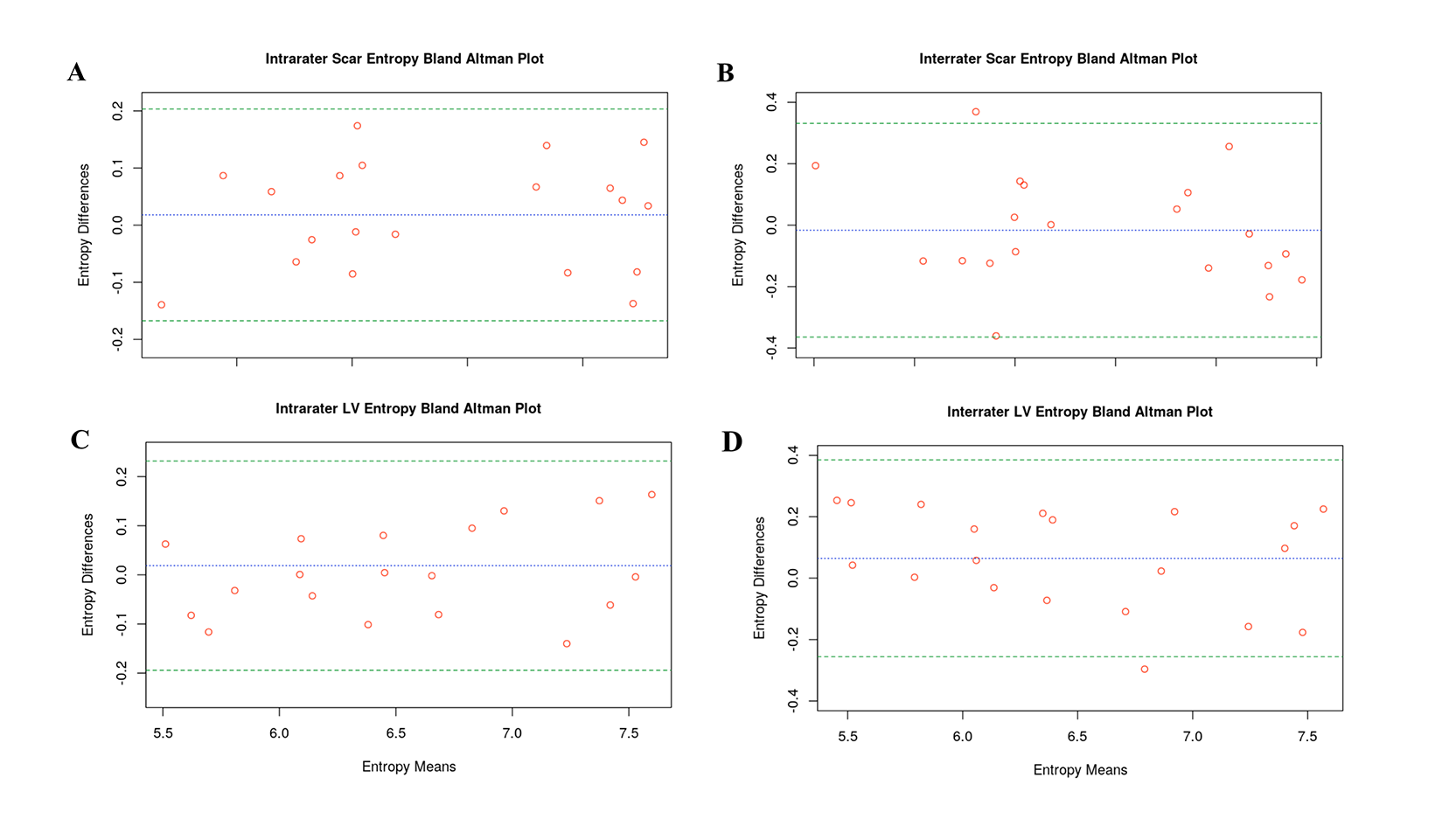

Supplement: Supplementary file 2 [file Image_1.TIF]
